# Editorial: Occurrence of harmful algal blooms and marine biotoxins

**DOI:** 10.3389/fmicb.2026.1794724

**Published:** 2026-02-10

**Authors:** Pierina Visciano

**Affiliations:** Department of Bioscience and Technology for Food, Agriculture and Environment, University of Teramo, Teramo, Italy

**Keywords:** cyanobacteria, diatoms, dinoflagellates, eutrophication, marine biotoxins

This Research Topic aimed to explore the global circulation of harmful algal blooms (HABs), i.e., phytoplanktonic toxin-producing microalgae as a consequence of climate change and anthropogenic pollution sources (Griffith and Gobler, 2020). The objective was to understand changes in seasonal patterns, the frequency and location of HAB hotspots, and the diversity of causative species. HABs can produce marine biotoxins, which are classified as either water-soluble biotoxins, such as saxitoxin and domoic acid (responsible for paralytic shellfish poisoning and amnesic shellfish poisoning, respectively), or lipophilic biotoxins (i.e., okadaic acid and dinophysistoxins), which are associated with diarrhetic shellfish poisoning (Annunziata et al., 2023). Among the latter group, *Dinophysis* species are dominant along the coasts of many European countries and may cause the closure of shellfish collection areas due to the presence of okadaic acid analogs. Vieira et al. investigated the cell density of *Dinophysis acuminata* in water samples taken weekly along the Portuguese mainland coast over a 13-year period from 2006 to 2018 and found that it was primarily favored by low sea surface temperature and high photosynthetically active radiation. These environmental factors were selected as conditions for producing empirical models capable of predicting the algal population sizes.

Phytoplankton communities are also influenced by nutrient composition, which varies under various dynamic processes, such as submarine groundwater discharge (SGD), sediment suspension events, and upwelling. Becerra-Reynoso et al. reported rapid increases in diatom populations due to new nutrients supplied to the system by upwelling. Conversely, when alternative nutrients, such as dissolved or particulate organic nitrogen, were dispersed into the environment through SGD, mixoplankton (e.g., dinoflagellates and other unknown flagellates) tended to predominate.

The toxicity of a new strain of *Karenia papilionacea*, isolated from the Yellow Sea in China, was assessed by exposing marine organisms to cell cultures of this algal species. Significant adverse effects were reported, with more than 50% mortality occurring within 48 h for rotifers and finfish, and after 72 h for brine shrimp. A low impact on the hatching success of healthy brine shrimp eggs was observed (Chen, Gao et al.).

Mi and Liu described the structure and properties, origin, and toxicity of tetrodotoxin (TTX), a lethal neurotoxin most commonly found in fish belonging to the *Tetrodontidae* family, but also in other marine organisms, such as mollusks, echinoderms, and snails (Hu et al., 2022). The authors also summarized the most recent analytical methods for TTX detection, such as bioassays, immunoassays, chromatographic analysis, and biosensors, including their advantages and limitations.

Harmful cyanobacterial blooms (cyanoHABs) are particularly abundant in warm, nutrient-rich freshwater lakes, reservoirs, and estuaries. They produce secondary metabolites called cyanotoxins, which can have potential adverse effects on animals, plants, and humans. The impact of seasonal hydrological disturbances on cyanoHABs in coastal Louisiana was studied by Hammond et al., who found higher biomass and maximum microcystin concentrations in summer and fall compared to winter. The influence of weather on phytoplankton, along with that of water temperature and salinity, was also reported.

Microcystin toxicity is associated with protein phosphatase inhibition and potential liver cancer (Schreidah et al., 2020). Several control strategies have been actively explored, such as the coculture of *Microcystis aeruginosa* with bacterial species belonging to the genera *Bacillus, Brevibacillus*, and *Chryseobacterium* (Zhang et al., 2021, 2019). It should be noted, however, that the use of algicidal bacteria can induce the release of harmful secondary metabolites, and therefore, it is necessary to select specific strains capable of degrading both algae and cyanotoxins (Massey and Yang, 2020). Chen, Xiong et al. isolated a *Bacillus subtilis* strain with strong algaecidal activity against *M*. *aeruginosa* and microcystin-degrading ability. This degradation process may occur primarily within bacterial cells and has been associated with the *mlr* gene cluster, which encodes a set of enzymes capable of breaking down microcystin molecules (Morón-López et al., 2023).

Similarly, the coculture of *M*. *aeruginosa* with the non-toxin-producing cyanobacterium *Synechococcus elongatus*, supplemented with 3-(3,4-dichlorophenyl)-1,1-dimethylurea as a photosynthesis inhibitor, reduced both algal biomass and microcystin production. This activity was more effective at 30 °C than at 23 °C and under high-nitrogen, low-phosphorus conditions (Lee et al.).

In conclusion, global warming, acidification, and deoxygenation of marine and freshwater ecosystems, which cause eutrophication and HAB phenomena, should be addressed to protect the environment, animals, and human health. Including HAB species in experimental studies and monitoring programs could provide a more ecologically relevant perspective within a One Health approach.
